# Pseudoinefficacy: negative feelings from children who cannot be helped reduce warm glow for children who can be helped

**DOI:** 10.3389/fpsyg.2015.00616

**Published:** 2015-05-18

**Authors:** Daniel Västfjäll, Paul Slovic, Marcus Mayorga

**Affiliations:** ^1^Department of Psychology, Linköping UniversityLinköping, Sweden; ^2^Decision Research, EugeneOR, USA; ^3^Department of Psychology, University of OregonEugene, OR, USA

**Keywords:** pseudoinefficacy, affect integration, singularity effect, prosocial behavior, psychic numbing, compassion

## Abstract

In a great many situations where we are asked to aid persons whose lives are endangered, we are not able to help everyone. What are the emotional and motivational consequences of “not helping all”? In a series of experiments, we demonstrate that negative affect arising from children that could not be helped decreases the warm glow of positive feeling associated with aiding the children who can be helped. This demotivation from the children outside of our reach may be a form of “pseudoinefficacy” that is non-rational. We should not be deterred from helping whomever we can because there are others we are not able to help.

“Beliefs of personal efficacy constitute the key factor of human agency. If people believe they have no power to produce results, they will not attempt to make things happen (Bandura, 1997, p. 3)”.

## Introduction

What motivates us to help others whose lives are endangered? More specifically, what motivates us to help in certain situations, while in others, we turn away? The answer to this question depends on the extent to which we value potential or actual losses of lives. Normatively, the scope or magnitude of a disaster or crisis should be the main carrier of value and motivation to act (Dickert et al., 2012). But, descriptively, our actions are sometimes insensitive to, or even demotivated by, increasing numbers of people at risk (Slovic, 2007). For example, a single identified victim often evokes stronger feelings and greater willingness to help than an unidentified single victim or a group of victims, identified or not. The preference for helping a single identified victim over a group of victims is known as the *singularity effect* (Kogut and Ritov, 2005b). In other circumstances, decision makers appear to be constructing their life-saving preferences on the basis of contextual information that may not be normatively justifiable. In a study by Jenni and Loewenstein (1997), participants evaluated a program saving two lives annually more favorably when those two lives were half of a population of four at risk, than when they were a much smaller percentage of 1,700 at risk. Slovic et al. (2002) termed this type of effect *proportion dominance* (see also Bartels, 2006), and argued that affective feelings play a central role in this phenomenon.

In the present article, we examine another contextual factor that may not be normatively justified: information about lives we cannot save may induce negative affect and demotivate us from saving those we can through dampening of good feelings.

### Pseudoinefficacy

Decisions are strongly motivated by perceived efficacy (Cryder et al., 2013; Erlandsson et al., 2013). Inefficacy, real or perceived, shrivels response, even among those who have the desire and the means to protect and improve lives. We propose that ineffectiveness is linked to negative affect and avoidance behaviors (Västfjäll and Slovic, 2013). It is especially troubling that efficacy sometimes goes unrecognized and vital aid that could be provided is withheld due to an illusion of ineffectiveness that we have named *pseudoinefficacy.*

In reviewing what appeared to be unrelated findings from two earlier studies of life-saving decisions, we uncovered a curious connection that motivated the present research. These prior studies asked people to provide clean water to aid people facing death from disease (Fetherstonhaugh et al., 1997) or to provide money to protect a child from starvation (Small et al., 2007). Fetherstonhaugh et al. (1997) found that people were less likely to send clean water that could save 4,500 lives in a refugee camp when the number of people in the camp was large (250,000) than when it was small (11,000). Small et al. (2007) found that the money donated to a 7-year-old African child facing starvation decreased dramatically when the donor was made aware that the child was one of millions needing food aid.

The findings from these two studies may have broad implications for prosocial or humanitarian behavior in light of the insights of Andreoni (1990), who contended that we help others not only because they need our help but because we anticipate and experience the *warm glow* of good feeling associated with giving aid. Subsequent empirical studies have supported this contention (e.g., Dunn et al., 2008). We hypothesize that knowledge of those “out of reach” (more in the large refugee camp and millions of starving people in Africa) may have triggered negative feelings that countered the good feelings anticipated from giving aid, thus demotivating action. A related explanation is that, compared to the large numbers of persons out of reach, the prospective aid created a sense of inefficacy, that is, a “drop-in-the-bucket” effect (Bartels and Burnett, 2011). Although the results from these studies by Fetherstonhaugh et al. (1997) and Small et al. (2007) may appear at first glance to reflect inefficacy, this is not really inefficacy, because the donor can actually help some people (from 1 to 4,500). Instead, it is a form of *pseudoinefficacy* that is non-rational. We should not be deterred from helping one person, or 4,500, just because there are others we cannot help.

In the present article we extend these findings to situations involving what we call fast or intuitive pseudoinefficacy. We propose that fast pseudoinefficacy is linked to virtually immediate dampening of warm glow by negative feelings, perhaps of sadness or unhappiness, in situations with *small numbers* of *identified* people in need and *small numbers unable to be helped*. We describe a series of studies designed to examine fast pseudoinefficacy and clarify the affective processes that contribute to it.

To test our hypothesis that, even when the numbers of affected individuals are small, negative affect associated with awareness of those not helped reduces the warm glow arising from doing good things, we employ a paradigm involving helping one or more starving children identified by name, age, photo, and so on. We systematically vary the number of children who can be helped and the number who cannot. A critical feature of our paradigm that distinguishes it from the typical design in previous proportion dominance studies, is that we inform participants that the children not helped should be irrelevant to the judgments about the child that could be helped (hence **pseudo**-inefficacy). However, as in previous proportion dominance studies, we expect that participants would pay attention to the children not helped, leading to decreased warm glow for the child that could be helped.

### The Affective Basis of Pseudoinefficacy

Affective feelings play a central role as a common currency, allowing decision makers to compare or integrate values for multiple, diverse stimuli (Cabanac, 1992). But sometimes the integration goes awry in a peculiar form of affective calculus that Polish poet Zbigniew Herbert has called “the arithmetic of compassion” (see, e.g., Herbert, 2007, p. 286). For example when only one life is at stake, the value attached to saving or prolonging that life is extreme. But as the number of lives at risk increases, phenomena such as psychophysical numbing and psychic numbing (Slovic, 2007), appear to lead our fast, intuitive, gut reactions on a path much different from one guided by the normal logic of arithmetic. With numbing, one life plus one life may be valued at something less than two lives. With compassion fade or collapse, 1 + 1 may be valued as less than 1!

Pseudoinefficacy is foreshadowed by a number of studies of affect integration in other domains. In impression formation, research shows that adding information that is moderately positive to information that is highly positive leads to lower judgments (Anderson, 1981), resulting from averaging values rather than adding them. An example of this from the consumer domain is provided by Yadav (1994), who asked consumers to rate their preference for different sets of furniture. Participants in the individual-item condition read information about a bed that pretest participants had rated as excellent. Those in the bundle condition rated a set consisting of two items: the same highly favorable bed plus a chest that was described as moderately favorable. Participants gave higher preference ratings to the bed alone than those in a separate group gave to a set containing both the bed and the moderately favorable chest (see also Weaver et al., 2012). Interestingly, Kralik et al. (2012) found evidence for similar averaging in non-human primates; rhesus monkeys preferred a high-value food item alone to the same item paired with one of positive but lower value. Similarly, Hsee et al. (1999) asked respondents to state the amount they were willing to pay to purchase each of two sets of dinnerware. Set S contained 24 pieces, all in good condition. Set J contained all of the same pieces plus eight more, all in good condition, along with 16 other pieces that were broken (40 total). In a single (separate) evaluation, respondents were willing to pay more for set S, though it was the inferior option, apparently devalued by the broken pieces. But in joint (side-by-side) evaluation, respondents were willing to pay more for Set J. Thus, in separate evaluation, negative affect appears to reduce positive affect through averaging.

Similarly, studies of the dilution effect compare the impact of diagnostic information with that of non-diagnostic information. The former is knowledge that is useful in making a particular judgment, whereas the latter is not relevant to that judgment. Research has shown that when both kinds of information are relevant, people tend to under-rely on diagnostic information (Nisbett et al., 1981; Tetlock et al., 1996). Thus the presence of non-diagnostic information weakens, or dilutes, the impact of diagnostic information.

Based on the above research, we hypothesize that the feelings and responses to children who can be helped may be dampened by being integrated with the lesser or even negative feelings associated with learning of children who cannot be helped.

Specifically, the present research set out to examine whether negative feelings coming from children that cannot be helped would dampen the positive feelings for the children, perhaps through some form of affective averaging.

## Experiments

### Overview

In Study 1, an initial test of pseudoinefficacy was conducted wherein participants rated their feelings and indicated how much money they would donate to either one child (who would be helped for certain) or *one* of two children (one child cannot be helped, but uncertain which child). In Study 2, we employed two experimental conditions where one child is helped and five other children cannot be helped. In the uncertain condition participants were not told which child of the six would be helped. In the certain condition participants were explicitly made aware of which of the children they could and could not help. Study 3 introduced ratings of warm glow. It included more variations of the number of children helped or not helped (always specifying which children could be helped, and which could not be helped). Studies 4a,b employed within-subjects designs where participants rated warm glow as a function of the number of children helped. Finally, Studies 5a,b tested whether including irrelevant neutral visual distractors or affect-inducing non-children pictures would produce dampening of warm glow for helping a child.

### Ethics Statement

Experiments were conducted in accordance with the ethical standards laid down in the 1964 Declaration of Helsinki. Studies were approved by the local ethics committes where the data was collected (Västra Götalands regional ethics board, Studies 1, 2, 5a,b), and IRB University of Oregon, Study 3, and 4a,b). Participants were compensated for their participation and gave their informed consent prior to inclusion in the studies. In all studies, participants received information about the study prior to participating. After completing their task, participants were thoroughly debriefed.

### Study 1: Initial Demonstration of Pseudoinefficacy

This initial study was designed to extend the large-number paradigms used in previous studies (Fetherstonhaugh et al., 1997; Small et al., 2007) by introducing a paradigm where participants are asked to donate to a either single child or one out of two children.

#### Method and Design

Ninety-four undergraduates (48 males) at Göteborg University, Sweden with a mean age of 27.3 (SD 5.1) participated in this study. A procedure devised by Small et al. (2007) was used in which participants, after completing an unrelated survey, received seven Swedish 10-kronor coins, a blank envelope, a questionnaire including the three response measures, and a charity request letter. The experimenter instructed participants to first read the charity request letter carefully, then place their donations (if any) in the envelope. Next, participants were asked to complete the questionnaire and return both the letter and questionnaire in the sealed envelope.

The letter informed the participant of the opportunity to donate any of their just-earned 70 kronors to the organization Save the Children. Participants were randomly allocated to one of the single or uncertain conditions. First, there were two single-child conditions: a description and picture of either a 7-year-old girl, Rokia (*n* = 21) or a 9-year-old boy, Moussa (*n* = 22). Participants were instructed that

Any money that you donate will go to Rokia [Moussa]. Rokia [Moussa] is desperately poor, and faces a threat of severe hunger or even starvation. Her [His] life will be changed for the better as a result of your financial gift. With your support, and the support of other caring sponsors, Save the Children will work with Rokia’s [Moussa’s] family and other members of the community to help feed her [him], provide her [him] with education, as well as basic medical care and hygiene education.

In the “uncertain” condition (*n* = 51), participants received a similar description but with pictures and stories of both Rokia and Moussa. Participants were instructed that their donations would go to Rokia *or* Moussa.

Three response measures were used:

(1) Willingness to donate. Participants could circle any number between 0 and 70 Swedish Crowns (SEK) in 10-crown increments.
(2) Affect. Participants rated “how do you feel about donating to Rokia/Moussa/the child?” on a scale ranging from *Negative* (-1) to *Positive* (+5).
(3) Perceived probability that the donation would make a real difference (1–5 scale anchored by *Not at all likely* to *Very likely*).

#### Results and Discussion

**Table 1** shows means comparing the uncertain condition to the average of the two single conditions. Independent *t*-tests showed that donations were significantly higher in the single conditions, *t*(92) = 2.12, *p* < 0.05. Affect ratings were also more positive in the single conditions, [*t*(92) = 2.46, *p* < 0.05]. The perceived probability of making a difference did not systematically differ between conditions, *t*(92) = 1.76, *p* = 0.08, but as can be seen in **Table 1**, there was a trend for higher probability in the single child condition.

**Table 1:** Willingness to donate, affect, and probability for uncertain and single conditions.

		Uncertain
		One of two
	Single child	children
	(*n* = 43)	(*n* = 51)
Donations (SEK)	26.5 (11.7)	20.2 (13.1)
Affect	3.7 (1.2)	3.1 (1.2)
Probability	2.4 (0.7)	2.1 (0.8)

SD in parenthesis.

Although donors may consider their contributions to be a drop in the bucket in some circumstances, such thinking is unlikely to underlie the results of this study, given that half of the two children at risk could be helped. Because the instructions in the uncertain condition explicitly stated that one of the two children would be helped, we believe that participants considered the one child not helped in the uncertain conditions and that this reduced affect and donations.

### Study 2: Comparing Uncertain and Certain Conditions Where Children are Explicitly Not Being Helped

It is possible that results from Study 1 were affected by the uncertainty regarding which child would be helped (Gneezy et al., 2006). Previous research has found that uncertainty elicits negative affect (Kurtz et al., 2007). In Study 2, we controlled for this possibility by directly contrasting an uncertain condition (one of *n* will be helped, but uncertain which one), and a certain condition (this child will be helped for sure; these others will not be helped).

#### Method and Design

One hundred and four undergraduates at Göteborg University, Sweden (65 females) with a mean age of 24.1 participated in Study 2.

We collected ratings of warm glow and affect in a between-groups design where participants either saw one child who could be helped (*n* = 37) or one of two scenarios where one child could be helped and six could not be helped. In the uncertain condition (*n* = 33) participants were not told which one of the seven children could be helped. In the certain condition (*n* = 34), the child who could be helped was identified.

In the single-child condition, an identified child with a photo and a name was presented. Participants were instructed that:

This is a picture of Nayani. Her living conditions are very bad and she needs your help. Without your help Nayani will likely not survive. You can help Nayani by donating money. Below are a number of questions about your thoughts and feelings about helping Nayani.

The certain condition included seven pictures, each depicting a named child. Participants were instructed that:

This is picture of some children. Their living conditions are very bad and they need your help. Without your help these children will likely not survive. You can help one of these children by donating money. The child you can help is located to the left. Her name is Nayani.

The presentation included identifying information about Nayani and a color photo, while photos of the six other children were faded and printed in black and white.

In the uncertain condition participants saw pictures of all seven children (all were named and presented in color) and were instructed that:

This is picture of some children. Their living conditions are very bad and they need your help. Without your help these children will likely not survive. You can help one of these children by donating money. Below are a number of questions about your thoughts and feelings about helping one of the children.

Participants in all conditions rated their feelings using three scales anchored by 0 (*Not at all*) to 7 (*Very much*):

(1) Warm glow: “If I donated money, I would experience a warm glow feeling.”
(2) Positive affect: “I have positive feelings when I think about Nayani/the child.”
(3) Compassion: “I have compassionate feelings for Nayani/the child.”

Participants were then debriefed and thanked for their participation.

#### Results and Discussion

Analysis of variances (ANOVAs) on the dependent measures yielded significant effects on all scales. Warm-glow, positive affect, and compassion ratings were significantly lower in the certain condition than in the single-child condition. The uncertain condition was not different from the certain condition on any of the three scales (see **Table 2**).

**Table 2:** Mean ratings and SD for warm glow, positive affect, and compassion for the three conditions in Study 2.

	One child	Uncertain	Certain	*F*	*p*
Warm glow	3.3_a_ (1.0)	2.9_a,b_ (1.1)	2.5_b_ (1.1)	4.46	<0.02
Positive affect	2.7_a_ (1.3)	2.2_a,b_ (0.8)	2.0_b_ (1.0)	3.28	<0.05
Compassion	4.1_a_ (1.1)	3.4_b_ (1.0)	3.2_b_ (1.4)	4.95	<0.01

SD in parenthesis. Subscripts not shared are significantly different at *p* < 0.05.

Together, these findings show that information about children not helped dampens affect for the child who could be helped. However, affect in the certain condition was not significantly lower than in the uncertain condition. These findings suggest that the negative feelings associated with awareness of the children not being helped dampen feelings for the child that could be helped. It should, however, be noted that the particular stimuli used here with the children not helped faded out may have dampened the emotional response to these children. In Study 3, a new paradigm was used where the children not helped were shown in a similar way to the child that could be helped. In addition, we further extended the “certain” paradigm from Study 2 to test how variations in the number of children helped or not helped influence warm glow.

### Study 3: Varying Numbers of Children Helped or Not Helped

#### Method and Design

**FIGURE 1:**
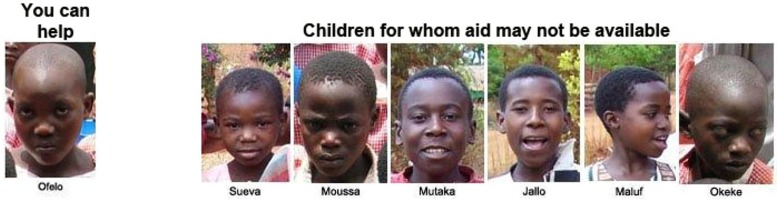
**Example of stimulus materials for one condition**.

Five hundred forty-three U. S. participants from a nation-wide sample (mean age 38, 56% female) completed an online version of the experiment. Study 3 directly examined the effect on warm glow of the number of children helped or not helped. Participants again saw pictures of children. In the first scenario seen by a participant we varied the number helped or not helped in a between-subjects design: 1/0 (helped/not helped), 1/1, 1/6, 2/0, and 2/1 (see **Figure 1** for an example of one condition). However, after seeing the initial scenario, participants in each condition saw all other scenarios in mixed orders, thus allowing us to also do an analysis of ratings within-subjects. The instructions read:

In this survey you will be presented with children in need of help. We will ask you to think about “warm glow,” a positive feeling that you may experience when you do something good for someone. Take a moment to think about one situation from your own life when you experienced this feeling. Next we will ask you to consider the warm glow you would expect to feel if you donated to help children in need.

Participants were then shown one of the conditions with a picture and names of the child(ren). For example, in the 1/6 condition they were instructed that:

These are seven children in need of aid. The child on the left, Ofelo, is facing starvation and is in immediate need of food. The six children on the right are facing ill health from water-borne diseases and are in immediate need of clean water and medicines. Suppose that you are now given the opportunity to donate money to a trusted aid organization to help Ofelo (the child on the left). Unfortunately, you can only help Ofelo and not the other children, for whom aid may not be available.

The warm-glow scale was an open-ended response anchored by 0 (*No warm glow*) to 100 (*Very strong warm glow*).

#### Results and Discussion

Means for both within- and between-subject comparisons are shown in **Table 3**. The overall between-subjects ANOVA was significant: *F*(1,540) = 2.39, *p* < 0.05. As can be seen in **Table 3**, for both between and within- subjects designs, warm glow associated with helping one child decreased linearly as the number of children not helped increased from zero not helped, to one not helped, and then to six not helped. Two children helped elicited warm glow similar to one child helped. A within-subject ANOVA (collapsing across different orders) also showed a significant condition effect, *F*(1,540) = 35.57, *p* < 0.0001. Comparing two helped and two helped/one not helped in the between–subjects design, a non-significant increase in warm glow occurred. However, within subjects, two helped/one not helped exhibited a statistically significant decrease in warm glow compared to two helped.

**Table 3:** Mean and SD for warm-glow ratings for both between- and within-subject comparisons (Study 3).

	Helped/could not be helped				
	1/0	1/1	1/6	2/0	2/1
	(*n* = 137)	(*n* = 100)	(*n* = 110)	(*n* = 86)	(*n* = 80)
Between-subjects	54.9_a_ (32.6)	50.8_b_ (31.3)	47.1_c_ (33.1)	55.8_a_ (31.0)	60.4_a_ (30.9)
Within-subjects (*n* = 502)	53.6_a_ (32.8)	49.6_b_ (31.8)	45.4_c_ (33.5)	53.7_a_ (32.2)	51.4_b_ (32.1)

SD in parenthesis. Mean not sharing a subscript are different at *p* < 0.05. Table entries are warm-glow ratings on a 0–100 scale.

Study 3 thus replicated the basic pseudoinefficacy effect and, further, showed that warm-glow ratings were sensitive to the number of children not helped. With the increasing number not helped, warm-glow feelings were further dampened. The effects were very similar for both within- and between-subjects comparisons. These results suggest that participants pay attention to magnitude and proportions even though changes in the number of children that could not be helped should be irrelevant to the feelings for the child that could be helped. This finding is thus largely consistent with what has been found in the literature on proportion dominance (Fetherstonhaugh et al., 1997). However, it is important to emphasize that while the reference group (the larger need) is relevant for the judgment in proportion dominance studies (saving 4,500 out of 11,000 feels better than saving 4,500 out of 250,000), our paradigm make explicit that the children not helped are “out of reach” and cannot be helped and thus should be irrelevant to the feelings for saving the child that can be saved.

### Studies 4a,b: Testing Robustness of the Effect in Within-Subjects Designs

Previous research (Hsee and Zhang, 2010; Kogut and Ritov, 2005a) has documented that in “joint evaluation” participants typically adjust their responses (so that they are the same) when they realize that their judgment is about the same object or stimulus (in this case the warm glow for a single child). The within-subjects results in Study 3, however, suggest that the pseudoinefficacy effect may resist this type of judgmental correction. In Study 4, we further tested this resistance by assessing warm glow in a within-subjects design with a fixed order of presentation of several different donation opportunities.

Study 4a was conducted as a classroom exercise and Study 4b was a replication using an individualized computer survey in a laboratory setting.

#### Method and Design

##### Study 4a

One hundred and forty-three students in a college classroom at the University of Oregon participated in this study. About two thirds were women. Their mean age was about 20. The number of children who could be helped was systematically varied in the following fixed order: six helped/one not helped, two helped/one not helped, one helped/one not helped, one helped. The general instructions were as follows:

Here are some questions about children in Africa who live in poverty. I will ask you to consider helping these children by donating money to a respected aid organization and then answer a number of questions about your thoughts and feelings.

In particular I would like you to think about warm glow—a positive feeling that you may experience when you do something good for someone. Have you experienced this? Take a moment and think about one situation from your own life when you experienced this feeling.

Next I will ask you to consider the warm glow you expect to feel if you donated to help children in need.

The first rating was for warm glow expected if one donated money to help six children (pictured, with names) but could not donate to a seventh, pictured and named child, as follows:

These are seven children in need of aid. Suppose that you are given the opportunity to donate money to a trusted aid organization to help the six children to the left (Nelson, Sueva, Moussa, Mutaka, Jallo, Maluf). Unfortunately you can only help these six children and not Okeke, for whom aid may not be available. Rate the warm glow you expect to feel if you donated money to help these six children (Nelson, Sueva, Moussa, Mutaka, Jallo, and Maluf).

Participants rated their warm glow by pressing a button on an audience-voting system (participants were instructed that the response options represented the following intervals on a 0–100 scale of warm glow: 1 = 0–20, 2 = 21–40, 3 = 41–60, 4 = 61–80, 5 = 81–100). Our data-collection method did not track individual answers, so only means are reported for this study.

##### Study 4b

Forty-eight University of Oregon undergraduates (mean age 20.5 years; 75% female) participated in a lab study. The methodology was similar to Study 4a, except participants rated the pictures in a computer survey. They responded using the same five-category scale of warm-glow used in Study 4a. Responses were tracked within-subjects, allowing statistical tests to be performed.

#### Results and Discussion

**FIGURE 2:**
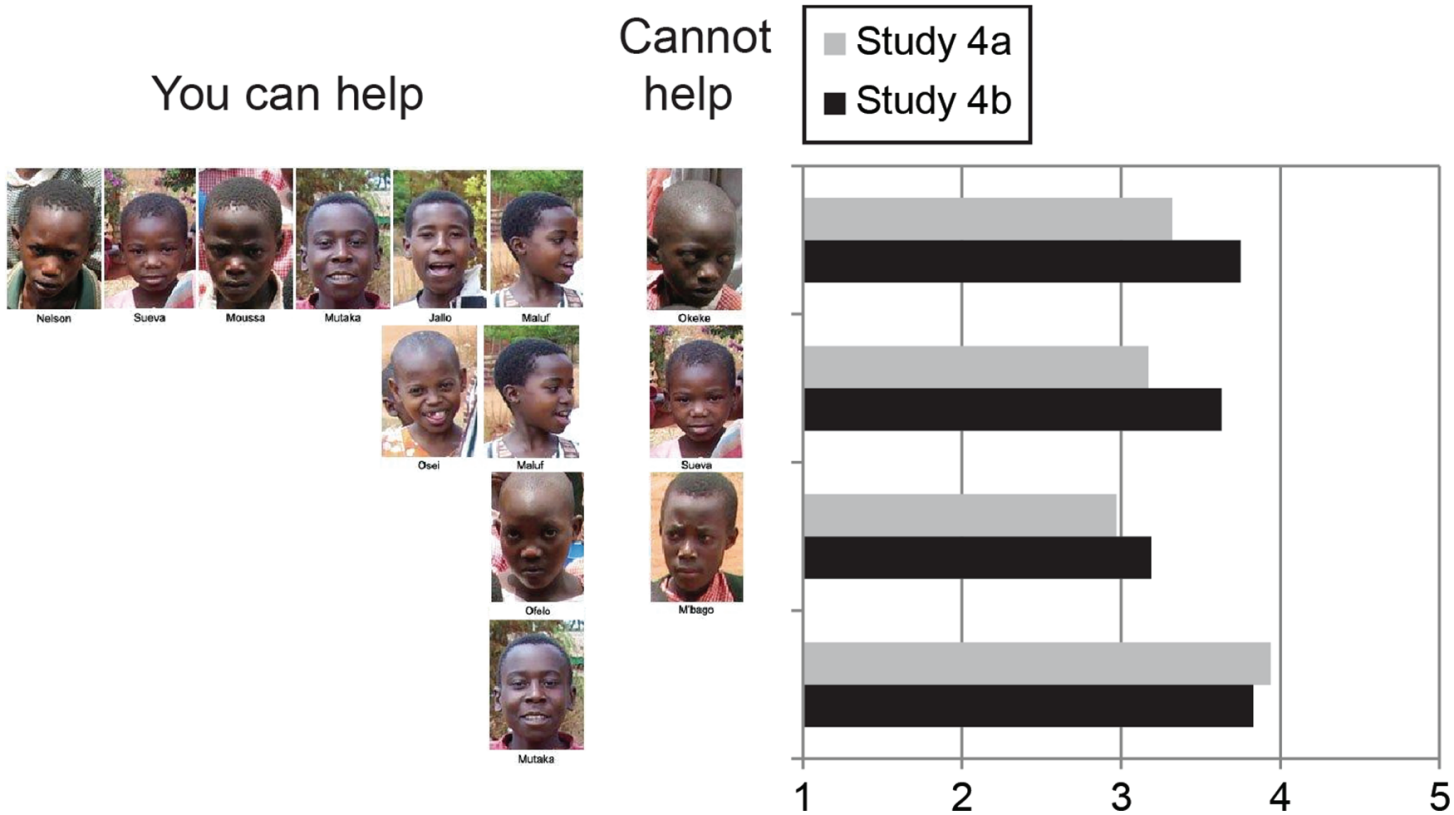
**Mean warm-glow ratings in within-subjects designs (Studies 4a,b)**.

In both Studies 4a,b, warm glow decreased as the number of children who could be helped decreased (see **Figure 2**). A within-subjects ANOVA showed a significant condition effect *F*(1,46) = 11.80, *p* < 0.01 for Study 4b. Importantly, the critical difference between one child helped/one not helped and one helped showed that the mean warm glow was substantially higher in the one-child condition. In Study 4b, this difference was significant in a Bonferroni *post hoc* test (*p* < 0.01).

Together, Studies 4a,b extended our earlier results in two ways: In contrast to the earlier studies, we kept the number of children not helped constant (one), and varied the number helped. We found that participants experienced less warm glow as the number of children helped decreased, a finding that is again consistent with proportion dominance (Fetherstonhaugh et al., 1997). However, the single child helped received the highest warm-glow rating consistent with the singularity effect (Kogut and Ritov, 2005a). Importantly, when the single child was paired with one other child not helped, that child then received the lowest warm-glow rating. It is notable that this effect occurred even with a fixed order where the single child appeared last and, arguably, participants would likely recognize that in both the one helped/one not helped and the one-helped scenarios they may help only one child and thus need not change their warm-glow ratings. We believe this is a demonstration of the robustness and pervasiveness of dampening of good feelings for the child one can help when paired with one or more children one cannot help. It should, however, be noted that the social setting that Study 4a was conducted in could have elicited other motives, such as reputation, that would have influenced warm glow feelings (Andreoni, 1990). However, given the similarity between Study 4a, conducted in a class room setting, and 4b, conducted as a more standard lab experiment, social esteem seemed not to drive the effect.

### Studies 5a,b: Testing an Alternative Explanation and a Possible Mechanism

Studies 5a,b had three goals; (1) provide evidence that the children not helped induce negative affect that dampens the positive affect for the child helped, (2) examine whether pseudoinefficacy could be produced by simply introducing other non-children stimuli, and (3) examine whether the role of negative affect could be isolated by incidentally manipulating negative affect.

In Study 5a we contrasted the effect on ratings of warm glow of children who cannot be helped with the effect of visual stimuli. If the dampening of warm glow observed in the previous studies was due to mere presence of additional visual objects while attending to the child that could be helped, we would also expect to find reduced warm-glow ratings in conditions with visual stimuli other than children.

In Study 5b we tested the hypothesis that a responsible mechanism for pseudoinefficacy is negative affect associated with the children not helped. We also compared the effect of children who cannot be helped with the effect of other visual stimuli that induce negative affect. We expected that other sorts of irrelevant pictures that induce negative affect would also reduce warm glow, consistent with our main explanation for the effect.

#### Study 5a: Non-Affective, Non-Child Stimuli

##### Method and design

**FIGURE 3:**
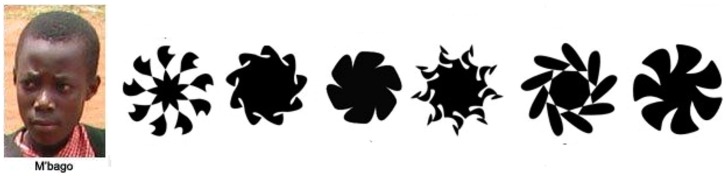
**Child and shapes used in the visual distraction experiment in Study 5a**.

One hundred and forty-eight undergraduates at Göteborg University, Sweden (mean age 32, 68% female) participated in an online survey. To test the effect on warm glow of visual distractors, we compared a condition (*n* = 54) where one child could be helped and six could not be helped with a condition (*n* = 44) where six shapes were substituted for the six children not helped (see **Figure 3**). In addition, a single-child condition was included (*n* = 44).

In each of the three conditions, participants were given the same general instructions to think about warm glow as were given in our prior studies and they were asked to rate the warm glow they expected to feel for the single child they could help, using a 0 *(No warm glow)* to 100 *(Very strong warm glow)* response scale.

Participants in the one/six children condition also rated how they felt when they viewed the children not helped (on a -2 to 2 scale from *very bad* to *very good*). Participants in the shapes condition similarly rated how bad or good they felt when they viewed the shapes on a four-point scale ranging from *Very bad* (-2) to *Very good* (+2).

##### Results and discussion

**FIGURE 4:**
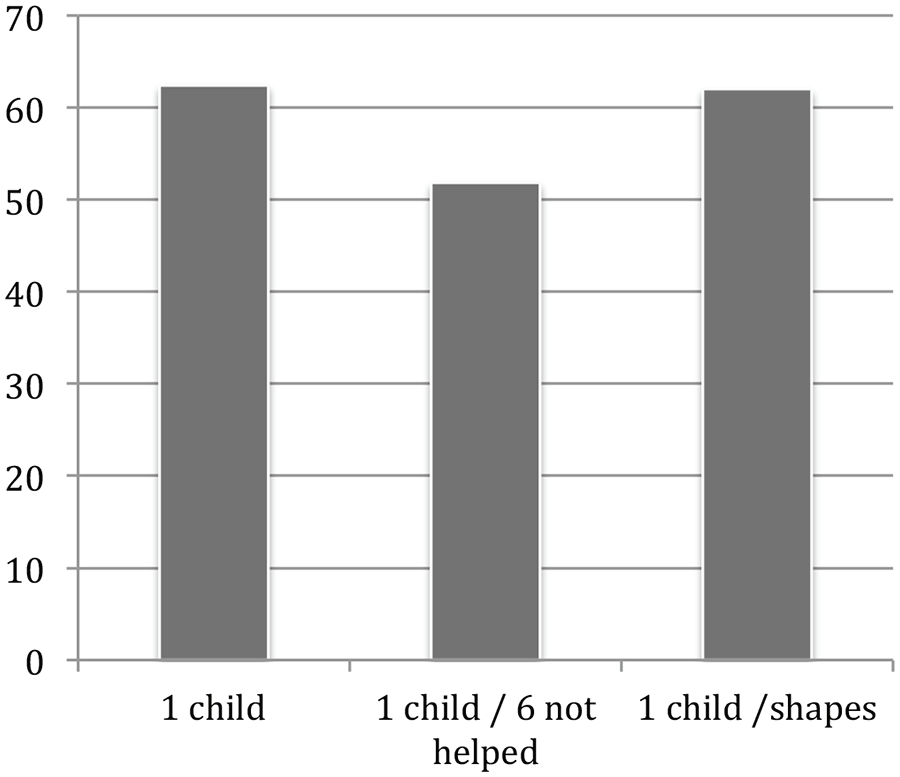
**Mean warm-glow ratings across the different conditions in the distractor study**.

An ANOVA showed a significant main effect of condition *F*(2,141) = 2.99, *p* = 0.05. As can be seen in **Figure 4**, mean warm glow was significantly lower in the one-child-helped/six-children-not-helped condition than in the single-child condition [planned contrast *t*(96) = 2.31, *p* < 0.03], replicating our previous findings. The one child/six shapes condition did not differ from the single-child condition [planned contrast *t*(96) = 0.91, *p* = 0.92], suggesting that visual distraction is not the cause of the observed pseudoinefficacy. Consistent with a negative-affect explanation, participants experienced significantly more negative affect (*M* = -0.28) when viewing the children not helped than when viewing the shapes (*M* = 0.31); [*t*(86) = 3.81, *p* = < 0.001]. Further, the correlation across participants between warm-glow ratings and the valence ratings of the children not helped was positive and significant, *r* = 0.32, *p* < 0.05, while the correlation between warm glow and valence ratings of the shapes was *r* = 0.00, *p* > 0.05.

#### Study 5b: Affective Non-Child Stimuli

In Study 5b we sought to determine the role of negative affect in dampening warm glow. The design was similar to Study 5a but, instead of affect-neutral shapes, pictures selected to induce strong negative affect were shown.

##### Method and design

**FIGURE 5:**
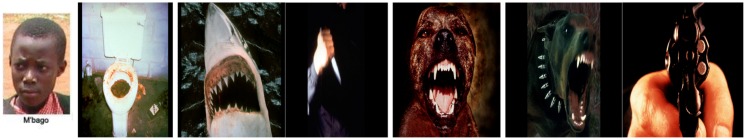
**Example of stimuli used in Study 5b**.

One hundred-four undergraduates at Göteborg University, Sweden with a mean age of 23 years (61% female) participated in a web-based survey. To test the effect of negative affect, we compared a condition (*n* = 26) where one child could be helped and six could not with a condition (*n* = 43) in which six negative affect-inducing pictures were substituted for the six children not helped (see **Figure 5**). The pictures were selected from the International Affective Pictures System (IAPS; Bradley and Lang, 2000). As in Study 5a, a single-child condition was included (*n* = 35). In addition to rating warm glow for the child, participants in the one-plus-six children condition rated how bad or good they felt when viewing the children not helped (-2 to +2 scale). Participants in the picture condition also rated how bad or good they felt when they viewed the set of pictures (-2 to +2).

##### Results and discussion

**FIGURE 6:**
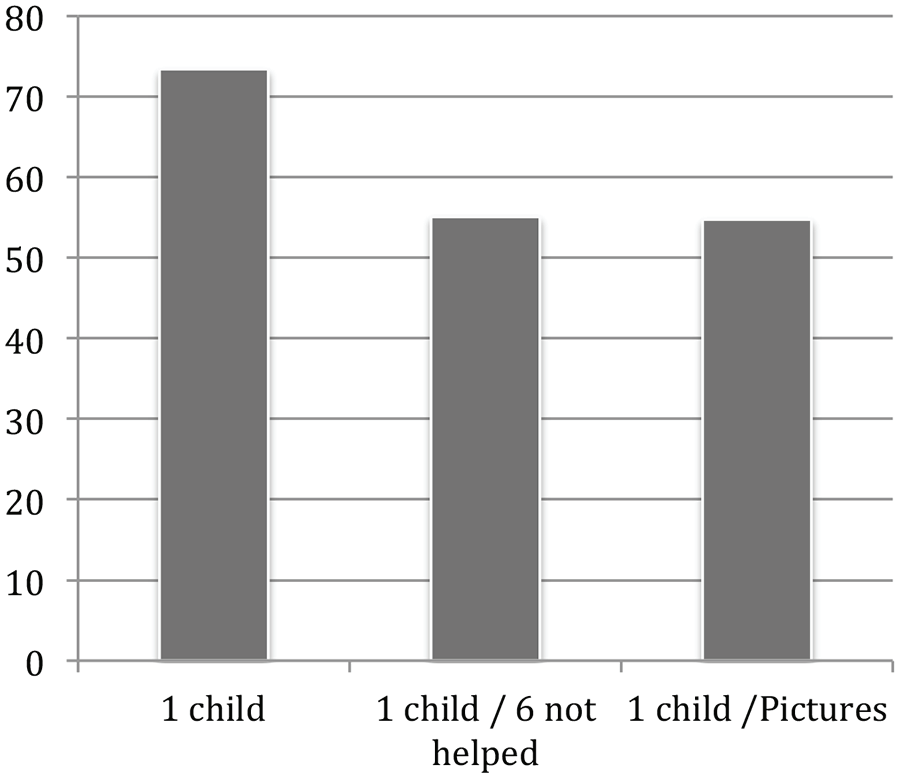
**Mean warm-glow ratings in the picture study (Study 5b)**.

Participants rated both the affect experienced while viewing the pictures (*M* = -0.53) and the children (*M* = -0.54) as negative. An ANOVA showed a significant main effect of condition on warm glow ratings, *F*(2,101) = 3.01, *p* = 0.05. As can be seen in **Figure 6**, mean warm glow was significantly lower in the one-child-helped/six-children-not-helped condition than in the single-child condition [planned contrast *t*(59) = 2.07, *p* < 0.05], replicating the basic pseudoinefficacy effect. Similarly, and critical to our hypothesis, warm glow in the one child/six pictures condition was also significantly lower than in the single-child condition [planned contrast *t*(76) = 2.27, *p* < 0.03], suggesting that irrelevant negative affect intruded upon and decreased the affect from helping the child.

Additionally, the correlation between warm-glow ratings and the valence ratings of the children not helped was positive and significant, *r* = 0.48, *p* < 0.05. Consistent with the affect explanation, the correlation between warm-glow and valence ratings of the pictures was similar in magnitude, *r* = 0.35, *p* < 0.05. Thus, both sources of negative affect, pictures of children not helped and diverse negative pictures, intruded upon and diminished anticipated warm glow from helping the child in need.

## General Discussion

The studies described here show that donors pay attention not only to relevant information (children who can be helped), but also incorporate feelings from normatively irrelevant information (e.g., children who cannot be helped) when that information is brought to their attention. Irrelevant negative feelings associated with those not able to be helped appeared to blend with the good feelings for those who can be helped, leading to dampened warm glow. This effect is not a form of inefficacy attributable to “drop-in-the-bucket” (Bartels and Burnett, 2011) cognitions because it occurs even when a substantial proportion of children, though not all, can be helped (e.g., see **Table 1**; **Figure 2**). The fact that one cannot help other children should not influence the decision to help a child who can be helped.

We demonstrated that fast pseudoinefficacy is an affective phenomenon—positive feelings about the child one can help are dampened by negative feelings associated with children who cannot be helped. In Studies 1–4, we found that affect ratings and feelings of warm glow (associated with the child one can help) were lower when children who could not be helped were made salient. In Study 5a,b, we demonstrated that the children not helped induced negative affect that reduced the positive warm glow for the child that could be helped. We also found that the pseudoinefficacy effect is not merely due to the presence of other stimuli. Warm-glow ratings of a single child who could be helped were not reduced when that child was accompanied by non-affective, non-children stimuli. In further support of an affect-based explanation, Study 5b showed that when other, unrelated, pictures that induced negative emotion accompanied the single child, warm-glow ratings were as low as in the pseudoinefficacy conditions where children not being helped were present.

While this research primarily documented that good feelings associated with helping decreased in presence of negative affect from children not helped, we believe that actual helping behavior is strongly motivated by these feelings. For instance, research has shown that both self-reported positive affect/warm glow as well as physiological indicators (facial muscle activity and activation of the reward circuitry in the brain) predicts if, and how much, people are willing to help (Genevsky et al., 2013; Västfjäll et al., 2014). Moreover, our Study 1 showed that hypothetical donations were lower (along with feelings of sympathy and compassion) in the pseudoinefficacy conditions.

The primary dependent variable in these studies has been positive affect/warm glow. We acknowledge that negative affect also may be a motivator of behavior (Manucia et al., 1984). For instance, guilt reduction predicts that individuals seek to repair their affective state by engaging in affect-enhancing activities such as giving (Cialdini et al., 1982; Clark and Isen, 1982). Further, the link between warm glow and giving in pseudoinefficacy contexts is not entirely clear from the present research. While previous research has suggested a close connection between anticipated warm glow of giving and the decision to donate (Andreoni, 1990), it is possible that both the decision to donate and the amount to donate may not show a 1-to-1 correspondence with warm glow (Dunn et al., 2008). Our results from Study 1 suggest a strong correlation between donations and positive feelings. But, since we only measured willingness to donate and positive affect simultaneously in our first study and then focused on the feeling component (positive affect/warm glow) in subsequent studies, an important task for future research is to examine this relationship more closely in the context of pseudoinefficacy.

Pseudoinefficacy has implications for theories of decision-making. With the common psychophysical form of numbing, the value attached to saving lives increases monotonically, but at a decreasing rate, as represented by the value function for gains in Prospect Theory (Kahneman and Tversky, 1979). With pseudoinefficacy, this monotonic increase can cease abruptly when one or more lives appear that are beyond help. For instance, in Study 4, warm glow increased monotonically with one, two, and then six children helped in the context of one that could not be helped. But the anticipated warm glow from helping a single child was far greater than the glow of helping one, two, or even six children in the presence of one out of reach, illustrating a version of what Markowitz et al. (2013) and Västfjäll et al. (2014) have termed *compassion fade*. Note also, in **Figure 2**, that the strong singularity effect exhibited for the child alone is greatly diminished when that child is accompanied by one child who cannot be helped. Perhaps this is a reverse singularity effect whereby the one out of reach creates a strong negative feeling that subtracts greatly from the warm glow. Future research should address this more closely.

Another important finding was the robustness of the pseudoinefficay effect. Earlier research in the domain of life-saving has shown that joint evaluation of options has been shown to reverse psychophysical numbing (Fetherstonhaugh et al., 1997). However, in Studies 4a,b, we found that pseudoinefficacy effects remained even in within-subject designs where people evaluated options in a joint evaluation mode. This finding is in contrast to most proportion dominance studies that find reliance on proportion in separate, but not joint, evaluation (but see Bartels, 2006). A striking example from Studies 4a,b is that warm-glow ratings for helping a single child were higher than in all conditions in which one child could not be helped, even though the single child to be helped was presented last. It can be argued that participants in our within-subjects studies should not be influenced by the children not being helped since the manipulation should be very transparent and salient. The fact that pseudoinefficacy remains in joint evaluation suggests that it is a robust phenomenon.

In summary, we use the term pseudoinefficacy to describe the attenuation of warm glow feelings for the child that can be helped, by presence or awareness of children that cannot be helped (and thus should be normatively unrelated to the judgment). These findings, together with previous research on proportional reasoning, suggest that there is a very close relationship between proportion of people helped and perceived efficacy. Further, proportion conveys affect, typically more strongly than does the absolute number helped (Slovic et al., 2002).

But why is proportion so important and so affective relative to absolute number, which should be the logical/rational driver of effectiveness? One could argue that it is because proportion cues perceived efficacy very strongly by making the absolute number of people helped, as well as the reference group easily accessible to people. Without the reference group, the affective meaning of the absolute number of people saved is less clear (Slovic et al., 2002). For example, Fetherstonhaugh et al. (1997) found that people’s willingness to intervene to save a stated number of lives was determined more by the proportion of lives saved than by the actual number of lives that would be saved. However, when two or more interventions were directly compared, number of lives saved became more important than proportion saved. Thus, number of lives saved, standing alone, appears to be poorly evaluable. With a side-by-side (joint evaluation) comparison, the number of lives became clear.

Slovic et al. (2002), drawing upon proportion dominance and the limited evaluability of numbers of lives, predicted (and found) that people, in a between-groups design, would more strongly support an airport-safety measure expected to save 98% of 150 lives at risk than a measure expected to save 150 lives. Saving 150 lives is diffusely good, hence only weakly evaluable, whereas saving 98% of something is clearly very good because it is so close to the upper bound on the percentage scale, and hence is readily evaluable and highly weighted in the support judgment. Subsequent reduction of the percentage of 150 lives that would be saved to 95, 90, and 85% led to reduced support for the safety measure but each of these percentage conditions still garnered a higher mean level of support than did the save 150 lives condition.

While the pseudoinefficacy effects documented here show a sensitivity to the number of children not helped, much like proportional reasoning, the fact that we informed participants that the children not helped *could not be helped* make our paradigm *conceptually* different from proportion dominance studies, where the reference group typically is normatively relevant to the judgment (Bartels, 2006). However, our results suggest that decision makers indeed pay attention to, and use available information about the reference group as if it were relevant for their judgment even in this context. Thus, proportion appears to be a primary driver of warm glow feelings and helping. But, interestingly, we also found dampening of warm glow even when a proportional judgment of numbers made no sense – pairing the child that could be helped with clearly irrelevant pictures (e.g., a shark) that induced negative affect, reduced warm glow. Pairing the child that could be helped with non-affective, non-children pictures did not decrease warm glow. Thus, it appears as if participants engaged in a form of affective proportional reasoning (good–bad judgments). Integration of irrelevant affective information in judgments likely occurs often since the affective system is poorly designed to judge true from false (Västfjäll and Slovic, 2013).

It should be noted that our pseudoinefficacy studies used small numbers (one to six) of identified victims who could not be helped. Future research should examine whether negative affect associated with large numbers of statistical victims who are out of reach (i.e., smaller proportion) demotivate individuals from helping in a similar way.

### Overcoming Pseudoinefficacy

Given that pseudoinefficacy appears to be a robust phenomenon with important implications, it is important to consider how it can be attenuated, eliminated, or reversed. It is typically the case that even our best efforts cannot help everyone in need. Thus it would be unfortunate, indeed, if we let this “incompleteness” deter us from accomplishing what is within our grasp.

But countering, or at least minimizing, pseudoinefficacy might not be easy. Kahneman (2011) summarizes a vast amount of research demonstrating that the human mind processes information in two ways: fast and slow (see also Kahneman and Frederick, 2002; Kahneman, 2003). Fast thinking, akin to what Haidt (2001) calls moral intuition when it comes to saving lives, is like perception. Moral feelings arise quickly and seem veridical, without reflection (Haidt, 2001), much like visual perceptions. But just as the human eye, as accurate as it is, can be deceived by certain patterns creating “visual illusions,” certain forms of contextual information, such as children who cannot be helped, may create “moral illusions.” And just as visual illusions may persist even when we know them to be false, the illusion of pseudoinefficacy may be similarly hard to dispel. In light of our findings, we can delete or minimize reference to the larger need the donation request addresses. One charity put the statistic, “3 million in need” above the picture of a starving child, likely demotivating many donors.

Since pseudoinefficacy appears to be an affective phenomena, perhaps a more promising strategy is the one used by Schwarz and Clore (1983) to block the intrusion of irrelevant feelings. Schwarz and Clore (1983) found that merely reminding respondents about the true source of their feelings (the weather) eliminated the affect-congruent influence on judgments (global well-being). Following, Schwarz et al. (2007), perhaps reminding participants that the source of the bad feelings they experience really is the children they *cannot* help, and not the child they can help, would eliminate pseudoinefficacy.

The strategy used by Schwarz and Clore (1983) works to alter the immediate feelings associated with fast thinking. Perhaps pseudoinefficacy can be overcome by teaching individuals to be compassionate and helpful through moral arguments (Haidt, 2001) or examples demonstrating illusions of non-efficacy in rather stark ways. For example, might a variation of Singer’s (2009) famous “child in the pond” example drive home the irrationality of pseudoinefficacy? Singer (2009) asks us to imagine ourselves walking past a shallow pond and seeing a small child playing in it suddenly slip under the water and begin to drown. “Would you, without hesitation, rush into the water to rescue the child?” he asks, “Of course you would,” he answers, and we nod in agreement. Consider the following addition to the story as a debiasing manipulation: “Now suppose, as you see the child go under, you also see, further away, another child begin to drown—one you cannot reach. Would you then be less motivated to rescue the child within your reach?” “Should you be?”

Perhaps a lesson in efficacy might also combat pseudoinefficacy. Consider the famous starfish story attributed to American author Eiseley (1969):

While wandering a deserted beach at dawn, stagnant in my work, I saw a man in the distance bending and throwing as he walked the endless stretch toward me. As he came near, I could see that he was throwing starfish, abandoned on the sand by the tide, back into the sea. When he was close enough I asked him why he was working so hard at this strange task. He said that the sun would dry the starfish and they would die. I said to him that I thought he was foolish. There were 1000s of starfish on miles and miles of beach. One man alone could never make a difference. He smiled as he picked up the next starfish. Hurling it far into the sea he said, “It makes a difference for this one.” I abandoned my writing and spent the morning throwing starfish.

## Conflict of Interest Statement

The authors declare that the research was conducted in the absence of any commercial or financial relationships that could be construed as a potential conflict of interest.
